# Rising and falling on the social ladder: The bidimensional social mobility beliefs scale

**DOI:** 10.1371/journal.pone.0294676

**Published:** 2023-12-05

**Authors:** Juan Matamoros-Lima, Guillermo B. Willis, Miguel Moya

**Affiliations:** 1 Mind, Brain and Behavior Research Center (CIMCYC), University of Granada, Granada, Spain; 2 Department of Social Psychology, University of Granada, Granada, Spain; University of Huelva: Universidad de Huelva, SPAIN

## Abstract

Recent works in the field of Social Psychology have shown the importance of studying subjective social mobility from different perspectives. In the literature about subjective societal mobility, most of the research is focused on the mobility-immobility framing. However, several authors suggested studying social mobility beliefs effects differentiating according to mobility’s trajectory, that is, upward (i.e., improving status over time) and downward (i.e., getting worse in status over time). The present research was motivated by the lack of measures that discriminate between beliefs in upward and downward societal mobility. Across two studies using different samples of the Spanish adult population, we examined both dimensions of social mobility beliefs and tested their predictive validity on other related constructs. In Study 1 (N = 164), with an EFA, we corroborated the independence between the two types of mobility. The internal structure was confirmed by a CFA in Study 2 (N = 400). Furthermore, it was shown that upward and downward mobility beliefs are differently related to other related constructs. The results from Studies 1–2 showed good convergent validity. In all correlations with the different constructs (attitudes towards inequality, meritocratic beliefs, justification of the economic system, and status anxiety) we found opposite direction effects for both types of societal mobility (upward and downward). The development of this new instrument can help to deepen our understanding of the psychosocial consequences of subjective social mobility, as well as to differentiate two processes that may have different consequences.

## Introduction

In objective terms, social class is usually determined by a combination of three types of resources [see e.g., 1–3]: economic, educational, and occupational prestige. However, social psychology has emphasized the importance of subjective perception when conceptualizing social class [4–6]. In this way, the psychosocial perspective takes as a reference previous studies carried out in other fields of social sciences and defines *social class* as “a stratification system based on access to resources such as wealth, property, power, and prestige [7, p. 9].

Social mobility—the possibility of moving from one social class to another—is a relevant social issue. According to the Organisation for Economic Co-operation and Development [8], around 40% of families in the OECD will remain in the same socioeconomic position from one generation to the next. However, most people still think that it is not difficult to climb up the economic ladder [9–11]. As such, there is an important gap between objective and subjective social mobility.

Most social psychological research about social mobility has focused on its objective dimension. These studies have operationalized social mobility as a change in income across one’s lifespan [12–14] or as a change toward more prestigious occupations [15,16]. However, the subjective dimension of social mobility has been less studied than objective, even though it has—by itself and independently of its objective counterpart—important social psychological consequences [17,18].

In addition, the few studies examining the social psychology of social mobility have used a mobility vs. immobility framework [19,20], without specifying whether the movement involves moving up to higher classes (i.e., upward mobility) or down to lower classes (i.e., downward mobility). As such, there is not a measure for discriminating between upward and downward social mobility beliefs. In this article, we aim to address this gap and present validity evidence for an instrument that discriminates between beliefs about upward and downward social mobility.

### Subjective social mobility

Social sciences have mainly emphasized the importance of the objective interpretation of social mobility [12,21,22]. However, in a complementary way, the psychosocial perspective has focused more on a subjective interpretation [10,11,23–27]. As such, whereas objective social mobility tends to be measured with indicators at the country level, such as the number of people moving from one social strata to the other [28,29]; subjective social mobility is measured by directly asking people to estimate the degree of mobility they think exists [10,23].

Subjective social mobility does not necessarily correspond to objective social mobility in the social structure, although an association between the two variables is frequently found [16,30–33]. Social psychology shows that subjective (vs. objective) reality is important in explaining human behavior [34,35]. For example, subjective socioeconomic status [36,37] and subjective economic inequality [38–40] have important consequences over and above their objective counterparts. Thus, subjective social mobility has important consequences that cannot be explained by objective social mobility [e.g., see 41,42].

In social psychology, subjective social mobility is understood as the belief about changes in status or social class over time [20]. Social mobility is not a unidimensional construct, and there is a need to study the possible effects of different types of social mobility [23,43]. Davidai and Wienk [44] argue that the different types of mobility depend on type (relative or absolute), time frame (past/current or future), trajectory (upward or downward), and target of comparison (personal or societal). In the present research, we will focus on subjective social mobility, that is, on beliefs about societal mobility (i.e., expectations about a change of status in society) differentiated according to the mobility’s trajectory: upward (i.e., improving status over time) or downward (i.e., getting worse in status over time).

When differentiating between upward and downward mobility, some studies suggest that there is an important bias in the subjective estimation of current social mobility. When thinking about overall social mobility, people tend to think more often about upward rather than downward mobility [45]. Likewise, Davidai and Gilovich [46] found that, when people assess the likelihood of moving along the social ladder, they perceive upward mobility as more likely than downward mobility.

Besides this bias toward upward social mobility, it is also important to differentiate between these two different types of mobility, as they may have different consequences. Schmidt [47], using a multilevel design including data from 21 countries, showed that experienced downward mobility positively predicted support for redistributive policies, while experienced upward mobility negatively predicted it. In the same vein, Mérola and Helgason [48] found in an experimental game that, when participants experienced an increase in income (i.e., upward mobility), they were less supportive of a tax increase; conversely, participants who experienced a decrease in income (i.e., downward mobility) were more supportive of a tax increase. Although the aforementioned studies examined the different consequences of upward and downward mobility, they did so by studying personal experienced mobility rather than beliefs about societal mobility.

In the literature about subjective societal mobility, most of the research is focused on the mobility–immobility framing [19,24]. Most studies examine the difference between those who believe that it is easy and those who believe that it is hard to change one’s economic status within a given society. But these studies do not make a clear distinction between the two possible types—upward and downward—of social mobility. Recently, some studies have suggested that, as it happens with personal experienced mobility [47,48], these two types of mobility can be considered relatively independent constructs [16]. For instance, in a study conducted in the United States, Browman et al. [23] found that an increase in perceived inequality decreased beliefs in upward mobility for poorer individuals and in downward mobility for richer individuals. In other words, participants perceived that, when wealth inequality is high, it is more likely that people will remain in their current economic positions.

Social mobility beliefs have been theoretically closely related to different constructs, such as meritocratic beliefs [the belief that getting ahead in society is based on talent and hard work; 49], the Protestant work ethic [the belief that hard work is a moral responsibility that allows one to achieve success; 50], and belief in a just world [the belief that people get what they deserve; 51]. Nevertheless, there is an important theoretical difference between social mobility beliefs and these constructs: social mobility beliefs refer only to movement (upward or downward) through different social positions in a predetermined social structure, regardless of the possible causes of the movement [e.g., through effort, talent, hard work, inheritance, or luck; 44] or the fairness of such movement [e.g., whether people got what they deserved; 24].

### Measuring subjective social mobility

Subjective social mobility has been operationalized as the subjective perception of the difference in one’s social status over time [15,42,52,53]. Thus, for instance, using a ladder scale with 10 rungs [36], participants estimate the difference between their socioeconomic status at two points in time (present vs. past or future). However, the results of some studies using this scale have found that people tend to place themselves in the middle points of the ladder [54,55], making mobility scores obtained with this instrument biased toward the midpoint.

Another way of measuring societal social mobility beliefs is by using numerical estimation of the percentage of people moving from one social stratum (e.g., quintile) to another [9,10,11,23,56]. However, people often have difficulty answering these questions and understanding the meaning of quintile or percentile [57–59], which prevents this from being a measure of social mobility with sufficient validity evidence.

Finally, different scales with Likert-type responses have been used to measure beliefs in social mobility [60–62]. However, these instruments do not distinguish between upward and downward social mobility. For instance, Browman et al. [19] used a unifactorial scale measuring social mobility beliefs that discriminates between high and low beliefs in mobility (or immobility) but does not differentiate the trajectory of mobility.

Taking the above into consideration, we consider it important to develop an instrument that can address the limitations mentioned. Using this new instrument, it would be possible to study the different types of subjective social mobility from a bidimensional perspective, discriminating between the different effects of upward and downward mobility.

### Overview of the current research

Through the present research, we have attempted to contribute to the study of social mobility from a bidimensional perspective, providing a new validated measurement instrument for studying the correlates of societal mobility beliefs according to their upward or downward trajectory.

For this purpose, we created an item pool about beliefs regarding social mobility (26 items), trying to collect items reflecting upward and downward trajectories [63,64]. A panel of experts evaluated different dimensions of the items (see supplementary material for details): *ambiguity*, *representativeness*, *intelligibility*, and *relevance* [65–68]. Then, across two studies we examined these two dimensions of social mobility and tested their predictive validity on other related constructs. In Study 1, we conducted an exploratory analysis to identify the factor structure of the item pool. In Study 2, we implemented confirmatory analyses to replicate the previous results. In addition, we tested the reliability of the measure and we analyzed the correlates between both dimensions of social mobility and related constructs.

The preregistration of Studies 1–2, all data code used, and supplementary material to this paper can be found available in the Open Science Framework (https://osf.io/7yqja/).

## Study 1

In Study 1, our main goal was to explore whether upward and downward social mobility are two independent and negatively related dimensions in social mobility (Objective 1). To achieve this objective, we carried out descriptive statistics of the items proposed to measure the two types of beliefs in social mobility and explored the factor structure of the scale. We also conducted an exploratory factor analysis and an internal consistency analysis.

Furthermore, to test the predictive validity of the scale, we explored the relationship between the two types of social mobility and other related constructs, such as subjective mobility and immobility (Objective 2) and support for economic inequality (Objective 3). In particular, we tested whether the new instrument measured different social mobility belief dimensions than the instrument developed by Browman et al. [19]. On the other hand, previous research has found a relationship between subjective social mobility and economic inequality. For example, Sharrif et al. [18], through an experimental study where societal mobility was manipulated through two conditions (high vs. low upward mobility), found that higher subjective upward mobility increased support for economic inequality. In other words, upward social mobility beliefs increase tolerance toward inequality. In this sense, we argue that, when people perceive that it is easy to move up on the social ladder (upward mobility), they will report positive attitudes toward economic inequality, whereas, when people perceive that it is easy to move down on the social ladder (downward mobility), they will report negative attitudes toward economic inequality.

## Method

### Participants and procedure

The survey was completed by 172 participants. Eight participants were excluded because they did not fulfill the preregistered inclusion criteria. The final sample (*N* = 164) consisted of 47.56% women (51.22% men and 1.22% other), with *M*_age_ = 43.41 years (*SD* = 12.34) and *M*_income_ = €5064.32 (*SD* = 12172.53). Most participants were in a relationship or married (76.22%), had an undergraduate or graduate education (80.48%), and worked full-time (75.46%; see S1 Table).

Data collection was carried out between Jun 08, 2021, and Jun 28, 2021. Data was reached online through social networks (e.g., Facebook, Twitter, etc.). Voluntary participation in the study was requested via text message targeted to social network users. The message consisted of a short text encouraging participation in a study on social issues and a link to access the survey. Participants gave their written consent to participate in the study, and the anonymity of their responses was guaranteed. There was no monetary compensation for participation in the study. The study was conducted after receiving approval from the Research Ethics Commission of the University of [blinded for peer review] (Date of approval: January 08, 2020; Approval Number: 969/CEIH/2019).

### Measures

#### Bidimensional Social Mobility Beliefs Scale (BSMBS)

Twenty items selected by a panel of experts were used (see supplementary material for more details) to assess beliefs about types of social mobility: upward and downward. Answers were provided on a 7-point Likert scale ranging from 1 (*totally disagree*) to 7 (*totally agree*).

#### Social Mobility Beliefs Scale (SMBS)

This scale is made up of eight items (own translation from 12) for measuring social mobility beliefs from a unidimensional perspective (mobility vs. immobility). Answers were provided on a 7-point Likert scale, ranging from 1 (*totally disagree*) to 7 (*totally agree*). Items included, for example, “Everyone, no matter who they are, can significantly change their status in society,” and, “People can substantially change their status in society.” High scores mean high beliefs in social mobility (α = .89).

#### Support for Economic Inequality Scale (SEIS)

Participants answered the Spanish version of the SEIS, with five items [Spanish adaptation by 69,70]. The response format was a 7-point Likert scale, ranging from 1 (*totally disagree*) to 7 (*totally agree*). The items included, for instance, “I am very concerned about the degree of economic inequality that exists in Spain today”. High scores mean high support toward economic inequality (α = .82).

#### Sociodemographic characteristics

Finally, we asked about some sociodemographic variables of participants: gender, age, nationality, marital status, educational attainment, occupation, participant’s income (calculated through a division of household income by number of members), subjective socioeconomic status [36], and political orientation (from 1 = *far left* to 7 = *far right*).

### Statistical analyses

To test whether beliefs in social mobility can be modeled by a two-dimensional (vs. one-dimensional) model and whether it shows evidence of internal validity, we conducted an EFA instead of other data reduction techniques, such as a principal component analysis. This was due to several key reasons. Firstly, the theoretical review and our empirical objectives turn towards modeling a two-dimensional model, that is, towards developing a measurement tool in which various indicators are intercorrelated with a smaller number of latent variables [71]. Secondly, EFA assumes a theoretical relationship between observed and latent variables [72–74]. Finally, since EFA and CFA are based on the common factor model [see 75], previous research suggests that EFA-based estimates are more likely to generalize to confirmatory factor analyses than principal component analysis [73].

A prerequisite for applying exploratory factor analysis is that the observed variables (items) are related to each other [72,76]. Therefore, we previously verified that the factor solution was interpretable using Bartlett’s sphericity and Kaiser-Meyer-Olkin index [77,78]. After that, we performed a parallel analysis to determine the number of factors to retain [79,80]. This is because it has been said of the Kaiser criterion (eigenvalues > 1) that “it can result in either overfactoring or underfactoring” [71, p. 23]. Finally, we conducted an exploratory factor analysis (EFA) using principal axis factoring because the normality assumption could not be corroborated, and oblique rotation (direct oblimin) due to the correlation between the factors was expected [71,72]. We used the *psych* package [81] to perform all the analyses mentioned. Analyses were carried out with R software [82].

## Results

### Exploratory factorial analysis

Both the Bartlett’s sphericity (Chi-square = 1771.58, df = 190, *p* < .001) and Kaiser-Meyer-Olkin index (.90), confirmed the relevance of this type of analysis. Horn’s parallel analysis [80] suggest retaining three factors accounting for 52% of the variance. Then, an oblimin rotation with a factorial solution restricted to three factors was performed. The first factor accounted for 26% of the variance, the second factor for 20%, and the third factor for 6%. Of the 20 items, 8 (loadings ≥ .65) were loaded on the first factor, 6 (loadings ≥ .50) were loaded on the second factor, 1 item were loaded both on the first and third factor, and 3 items were loaded both on the second and third factor. Two items did not load on either of these factors because their weights were lower than .3. No items were charged only in the third factor.

According to our purpose of developing a bifactorial scale that discriminates between upward and downward social mobility beliefs, we decided to eliminate items that 1) showed a high mean compared to the mean of its factor (see Table 1); 2) did not load on any of the three factors; 3) loaded simultaneously on different factors (see Table 2).

**Table 1:** Descriptive statistics of items (Study 1).

Item label	M	SD	Skewness	Kurtosis
**Upward social mobility **				
BSMBS_1u	3.74	1.68	0.08	-0.91
BSMBS_2u	4.34	1.60	-0.40	-0.52
BSMBS_3u	3.81	1.64	0.13	-0.78
BSMBS_4u	3.82	1.50	-0.01	-0.65
BSMBS_5u	3.77	1.50	-0.29	-0.73
BSMBS_6u	3.56	1.67	0.11	-0.94
BSMBS_7u	5.40	1.47	-1.03	0.71
BSMBS_8u	3.41	1.55	0.30	-0.42
BSMBS_9u	4.18	1.53	-0.09	-0.71
BSMBS_10u	4.02	1.55	0.05	-0.68
**Downward social mobility **				
BSMBS_11d	3.65	1.66	0.16	-0.94
BSMBS_12d	3.40	1.57	0.34	-0.41
BSMBS_13d	3.13	1.47	0.36	-0.52
BSMBS_14d	3.47	1.50	0.22	-0.62
BSMBS_15d	3.79	1.55	0.38	-0.49
BSMBS_16d	3.22	1.37	0.22	-0.38
BSMBS_17d	2.65	1.66	1.10	0.45
BSMBS_18d	3.12	1.48	0.43	-0.33
BSMBS_19d	3.31	1.51	0.51	-0.23
BSMBS_20d	3.76	1.64	0.1106	-0.829

*Note*: N = 164; M, mean; SD, standard deviation.

**Table 2:** Loadings of bidimensional social mobility beliefs 20 items scale (Study 1).

Items	F1	F2	F3	*h* ^*2*^
**BSMBS_8u**	0.81			0.61
BSMBS_1u	0.79			0.66
BSMBS_6u	0.76			0.61
BSMBS_3u	0.76			0.59
**BSMBS_9u**	0.74			0.63
BSMBS_5u	0.71		-0.32	0.60
**BSMBS_4u**	0.68			0.55
**BSMBS_10u**	0.66			0.67
BSMBS_2u	0.65			0.53
**BSMBS_14d**		0.75		0.54
**BSMBS_18d**		0.75		0.61
BSMBS_20d		0.73		0.59
**BSMBS_13d**		0.70		0.50
**BSMBS_11d**		0.62		0.41
BSMBS_19d		0.53	0.30	0.44
BSMBS_16d		0.50		0.49
BSMBS_7u				0.20
BSMBS_17d				0.04
BSMBS_15d		0.36	0.61	0.60
BSMBS_12d		0.35	0.51	0.52

*Note*: N = 164; F, factor; *h*^*2*^, communality; Standardized loadings > .30 are reported; in bold the items of the final scale.

After that, we performed a second Horn’s parallel analysis [80], which suggested retaining only two factors accounting for 55% of the variance. Then, an oblimin rotation with a factorial solution restricted to two factors was performed again. The first factor accounted for 30% of the variance and the second factor for 25%. Of the 14 items, 7 (loadings ≥ .67) were loaded on the first factor, 7 (loadings ≥ .60) were loaded on the second factor.

### Descriptive statistics and discrimination and reliability index

In order to explore whether the set of items selected for each factor presented a high discriminatory capacity, the discrimination index for each item was calculated [65]. For this purpose, the corrected correlation coefficient between the item score and the total score of the item’s factor of belonging was carried out. Values with a deviation equal to or greater than +/- .30 were considered adequate [83]. In addition, we calculated the difference between the correlation item- belonging factor and item-opposite factor [84]. Items with differences between the correlations lower than .15 were eliminated. Finally, to explore the homogeneity of the factors, the mean inter-item correlation was carried out [85] and the Cronbach’s reliability index was calculated. Considering the results of the above analysis, 6 items were eliminated: items 1, 3, 6, 16, 19, and 20.

In the version of the 8-items scale, the corrected item-total correlation in all the items was greater than .67. This result was observed for both upward social mobility (between .74 and .76) and downward social mobility (between .68 and .72) responses. Differences between the correlation item-belonging factor and item-opposite factor ranged from .16 to .33 for upward mobility items and .13 to .27 for downward mobility items. The mean inter-item correlation for each factor ranged from .58 to .60 for upward mobility and .51 to .54 for downward mobility, with Cronbach’s alpha > .80 for both social mobility beliefs (upward: α_Cronbach_ = .85; downward: α_Cronbach_ = .81). The overall mean for upward mobility was 3.86 (*SD* = 1.28), while for downward mobility it was 3.34 (*SD* = 1.22).

### Exploratory analysis

A bivariate Pearson correlation between upward and downward social mobility beliefs and other variables included in this study was carried out. As shown in Table 3 upward social mobility beliefs correlated positively with social mobility beliefs scale (*r* = .53, *p* < 0.001), and support for economic inequality scale (*r* = .32, *p* < 0.001). Downward social mobility beliefs presented opposite direction correlations, that is, it correlated negatively with social mobility scale (*r* = -.45, *p* < 0.001), and support for economic inequality scale (*r* = -.30, *p* < 0.001).

**Table 3:** Correlations coefficients between upward and downward social mobility beliefs and other constructs (Study 1).

* *	M	SD	USM	DSM	SMBS	SEIS	SSS
USM	3.86	1.28					
DSM	3.34	1.22	-0.53***				
SMBS	4.07	1.33	0.53***	-0.45***			
SEIS	1.86	1.10	0.32***	-0.30***	0.37***		
SSS	5.93	1.45	0.28***	-0.33***	0.41***	0.26**	
PO	2.74	1.24	0.45***	-0.38***	0.40***	0.43***	0.21**

*Note*: N = 164; USM, Upward Social Mobility; DSM, Downward Social Mobility; SMBS, Social Mobility Beliefs Scale; SEIS, Support for Economic Inequality Scale; SSS, Subjective Socio-economic Status; PO, Political Orientation; M, mean; SD, standard deviation

*p < 0.05

**p < 0.01

***p < 0.001.

To examine whether our scale measured a different type of mobility than Browman et al. [19] scale, we also ran an exploratory analysis with all items from both scales. Horn’s parallel analysis [80] suggested retaining three factors. Then we performed an oblimin rotation with a factorial solution restricted to three factors. The three factors were: Factor 1, items from Browman et al. [19] scale; Factor 2, items related to the upward mobility factor; Factor 3, items related to the downward mobility factor (see S2 Table).

## Discussion

The results showed that beliefs about upward social mobility can be considered different from those about downward social mobility (Objective 1). Regarding the internal structure observed through the EFA, it would be necessary to confirm it in an independent sample. On the other hand, exploratory analyses seem to suggest that the SMBS proposed by Browman et al. [19] appears to measure beliefs in upward social mobility (vs. immobility), rather than downward mobility (Objective 2) and that there is an inverse relationship between both types of social mobility and support for economic inequality, positive for upward mobility and negative for downward mobility (Objective 3).

## Study 2

In Study 2, we aimed to corroborate the bifactorial structure of the social mobility scale using an independent sample from Study 1. Regarding the predictive validity of the scale, we examined the relationship between beliefs about upward and downward social mobility, ideologies, and perceived threat.

Recent studies suggest that ideological beliefs may play a role in legitimizing the status quo [49]. These variables may have a palliative effect on the distress derived from perceiving the world as unfair [86,87]. Likewise, believing that it is possible to move up in society could increase support for these ideological beliefs.

On the contrary, perceiving downward mobility could decrease the defense of these legitimizing myths. For example, people who believe that their social position may worsen (vs. improving) in the future display lower perceived control over their lives [88]. In this sense, people who perceive high downward mobility may believe that they can lose their position on the social ladder, which, as a result, increases their status anxiety. On the contrary, perception of high upward mobility could decrease status anxiety.

To summarize, we expected that upward social mobility beliefs would be positively associated with meritocratic beliefs and economic system justification, whereas downward social mobility beliefs would be negatively associated with meritocratic beliefs and economic system justification. For perceived threat variables, we expected that upward social mobility beliefs would be negatively associated with status anxiety, while downward social mobility beliefs would be positively associated with status anxiety.

In the preregistration plan, we preregistered that there will be a difference in how strong these associations are (i.e., we predicted both the direction and the strength of the associations). Given that these hypotheses are not one of the main points of the present manuscript, which is focused on presenting the validity evidence of the Social Mobility Scale, we present these analyses in the Supplementary Materials.

## Method

### Participants and procedure

The survey was completed by 414 participants. Based on the preregistered inclusion criteria, 14 participants were excluded. The final sample was composed of 400 participants. The sample consisted of 60.75% women (38.25% men and 1% other), with *M*_age_ = 32.50 years (*SD* = 14.05), and *M*_income_ = €2863.01 (*SD* = 5879.58). Most of the participants were married or in a relationship (54%), had a university or postgraduate education (58.5%), and worked full time (44%; see S1 Table).

Data collection was carried out between Nov 16, 2021, and Dec 30, 2021. Data was reached online through social networks (e.g., Facebook, Twitter, etc.) and the institutional mail of a university in southeast Spain. Voluntary participation in the study was requested via text message. Participants gave their written consent to participate in the study, and the anonymity of their responses was guaranteed. Participants in the study were entered into a €50 prize draw among all participants. The study was conducted after receiving approval from the Research Ethics Commission of the University of [blinded for peer review] (Date of approval: January 08, 2020; Approval Number: 969/CEIH/2019).

### Measures

#### Bidimensional Social Mobility Beliefs Scale (BSMBS)

To validate the BSMBS, we used the eight items of the BSMBS after analyzing the data obtained from Study 1 (e.g., “In Spain, children often achieve a higher socioeconomic status than the household in which they grew up”; “The children of Spanish people come to belong to a higher social class compared to the class they come from”; “In Spanish society, most people have lower incomes from one generation to the next”; “The majority of Spanish families have lower social positions than the previous generation”; see S1 Appendix). High scores on upward/downward social mobility mean high beliefs in the different types of mobility.

#### Meritocratic Beliefs Scale (MBS)

Participants responded to the Spanish version of the MBS [Spanish adaptation by 89,90]. The response format was a 7-point Likert scale, ranging from 1 (*totally disagree*) to 7 (*totally agree*). This scale is composed of six items (e.g., “People who work hard do achieve success”, “If people work hard, they do get what they want”). High scores mean high meritocratic beliefs (α = .92).

#### Economic System Justification Scale (ESJS)

To measure this construct, we used the Spanish version of the ESJS [Spanish adaptation by 91,92], composed of seven items. The response format was a 7-point Likert scale ranging from 1 (*totally disagree*) to 7 (*totally agree*). Examples of items are: “The gap between social classes reflects differences in the natural order of things” and “It is good to have an economic system that rewards those who make an effort”. High scores mean high economic system justification (α = 0.83).

#### Status Anxiety Scale (SAS)

We used the Spanish version of the SAS [Spanish adaptation by 93,94]. The response format was a 7-point Likert scale, ranging from 1 (*totally disagree*) to 7 (*totally agree*). This scale is composed of five items (e.g., “I worry that my social status will go down,” “I worry that my current social status is too low”). High scores mean high status anxiety (α = 0.87).

#### Sociodemographic characteristics

Finally, we asked about some of the participants’ sociodemographic variables: gender, age, nationality, marital status, educational attainment, occupation, participant’s income (calculated through a division of household income by number of members), subjective socioeconomic status [36], and political orientation (from 1 = *far left* to 7 = *far right*).

### Statistical analysis

We conducted a confirmatory factor analysis (CFA) with the aim of exploring whether the dimensional structure observed in Study 1 was replicated. For that purpose, we used the *lavaan* package [95]. Considering non-independence of observations as well as the possible non-normality of the data, we used the robust maximum likelihood (MLR) estimation [96]. The model fit was assessed with the root mean square error of approximation (RMSEA) with a 90% confidence interval (CI), standardized root mean square residual (SRMR), Tucker–Lewis index (TLI), and the comparative fit index (CFI). RMSEA and SRMR values less than .06 and TLI and CFI values greater than .95 indicate good model fit [71]. Two different models were tested (see Table 4): a unifactorial model (Model 1), composed of one factor of social mobility; and a bifactorial model (Model 2), composed of two factors of social mobility (i.e., upward and downward social mobility). Also, we explored the homogeneity of the observed variables (items) in relation to the latent variables (factors) of belonging. Following this goal we performed a descriptive analysis of each item and assessed its discrimination index with the corrected item–total correlation method [65] using the *psych* package [81]. Analyses were carried out with R software [82].

**Table 4:** Confirmatory factor analysis of the bidimensional social mobility beliefs scale (Study 2).

Models	Chisq	df	p-value	CFI	TLI	SRMR	RMSEA [90% CI]
Model 1	297.497	20	0	0.63	0.48	0.11	0.18 (.16, .20)
Model 2	30.070	19	0.051	0.98	0.97	0.03	0.03 (.01, .05)

*Note*: N = 400; CFI = Comparative fit index; TLI = Tucker-Lewis index; SRMR = Standardized Root Mean Square Residual; RMSEA = root-mean-square error of approximation; CI = confidence interval; Model 1 = one factor of social mobility. Model 2 = two-factors, composed by upward and downward social mobility.

## Results

### Confirmatory factorial analysis

**Fig 1:**
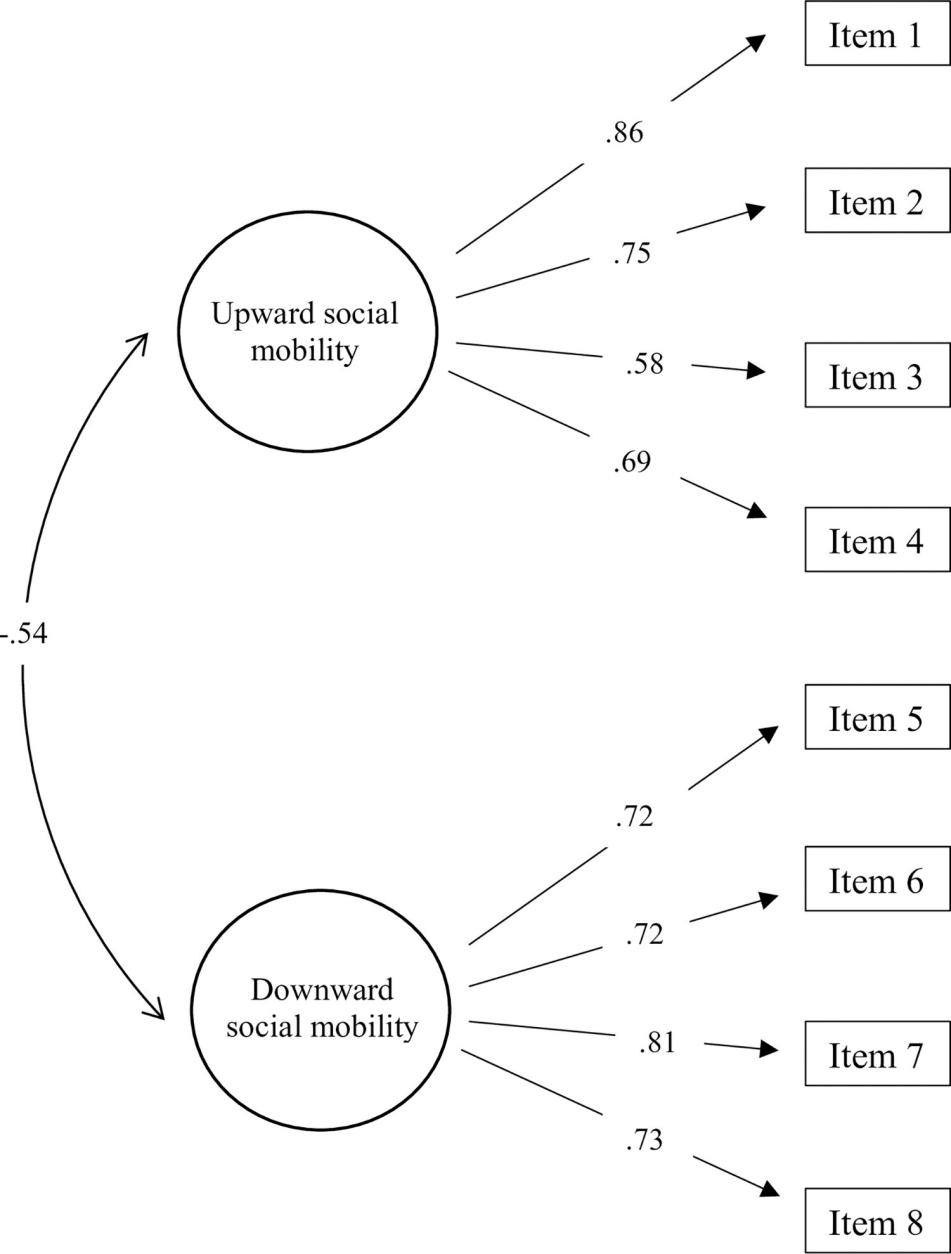
Dimensions of bidimensional social mobility scale (standardized factor loading).

Firstly, unifactorial and bifactorial models were compared and, we found significant differences between both models (χ^2^ = 161.85, df = 1, *p* < .001). Then, CFA confirmed that a bifactorial model (i.e., upward and downward social mobility) showed the best model fit in the assessed sample (see Table 4). A bifactorial model including 8 items (4 items per upward factor and 4 items per downward social mobility) was specified. All items have factor loading >.68 (see Fig 1). The results revealed a good fit of the bifactorial model: χ^2^ = 30.070, df = 19, *p* = .051; CFI = .98; TLI = .97; SRMR = .03; RMSEA = .03, 90% CI (.01, .05). Internal consistency was adequate (Upward: α_Cronbach_ = 0.81; ω_MacDonald_ = 0.81; r_meaninter-item_ = 0.52); Downward: α_Cronbach_ = 0.83; ω_MacDonald_ = 0.83; r_meaninter-item_ = 0.55). To provide a more robust of the structure and stability of BSMBS, we also explored the invariance based on different variables. We examined configural, metric, scalar, and residual invariance across gender (Male, Female) and Subjective Socioeconomic Status (“≤ 5” = Low SSS; “≥ 6” = High SSS). The results showed a good fit of configural, metric, scalar, and residual invariance across Gender (see S6 Table), and Subjective Socioeconomic Status (see S7 Table).

### Descriptive statistics and discrimination and reliability indices

All items showed adequate results in the discrimination index (see S10 Table): corrected item-total correlation (upward: ≥ .58; downward: ≥ .70). All standard deviations were higher than 1.2. Participant’s mean score on the scale was, 3.85 (*SD* = 1.09) for the upward social mobility, and 3.43 (*SD* = 1.13) for the downward social mobility.

### Corroboration of hypotheses

A bivariate Pearson correlation between the scores in the upward and downward social mobility beliefs and those obtained in the other variables included in this study was carried out. As shown in Table 5, upward social mobility beliefs correlated positively with meritocratic beliefs (*r* = .53, *p* < 0.001), economic system justification (*r* = .43, *p* < 0.001) and negatively with status anxiety (*r* = -.17, *p* < 0.001). Downward social mobility beliefs presented the opposite direction effect correlations, that is, it correlated negatively with meritocratic beliefs (*r* = -.24, *p* < 0.001) and economic system justification (*r* = -.18, *p* < 0.001), and positively with status anxiety (*r* = .26, *p* < 0.001).

**Table 5:** Correlations coefficients between upward and downward social mobility beliefs and other constructs (Study 2).

* *	M	SD	USM	DSM	MBS	ESJS	SAS	SSS
USM	3.85	1.09						
DSM	3.43	1.13	-0.44***					
MBS	2.94	1.42	0.53***	-0.24***				
ESJS	2.96	1.14	0.43***	-0.18***	0.79***			
SAS	4.24	1.51	-0.17***	0.26***	-0.13**	-0.03		
SSS	5.74	1.37	0.32***	-0.13**	0.26***	0.26***	-0.19***	
PO	2.71	1.22	0.26***	-0.06	0.53***	0.59***	0.04	0.15**

*Note*: N = 400; USM, Upward Social Mobility; DSM, Downward Social Mobility; SMS, Social Mobility Scale; MBS, Meritocratic Beliefs Scale; ESJS, Economic System Justification Scale; SAS, Status Anxiety Scale; SSS, Subjective Socio-economic Status; PO, Political Orientation; M, mean; SD, standard deviation

*p < 0.05

**p < 0.01

***p < 0.001.

## Discussion

The results replicated the internal structure of the BSMBS. The use of CFA adds empirical evidence to the conceptualization of both types of social mobility (upward and downward) as different entities. Furthermore, the results established a different relationship pattern among both types of social mobility and meritocratic belief, economic system justification ideology, and status anxiety. Our goal was to develop a measure that differentiates between upward and downward mobility instead of merely differentiating between mobility and immobility. Existing validated scales have treated mobility as a single construct. From this last perspective, it could be assumed that both types of mobility (upward and downward) have the same relationship with other variables. But we have shown that they do not: Upward is positively correlated with meritocracy and economic system justification, whereas downward is negatively correlated with it; the opposite is true when considering status anxiety.

## General discussion

The present research was motivated by the lack of measures that discriminate between beliefs in upward and downward societal mobility. Two studies showed that social mobility beliefs is a variable composed of two different dimensions: upward and downward mobility. In Study 1, with an EFA using a pool of 20 items, we corroborated the independence between the two types of mobility. This result was confirmed by a CFA in Study 2. Furthermore, we showed that upward and downward mobility beliefs are differently related to other related constructs.

We also showed that the BSMBS discriminates between two types of beliefs in societal mobility according to its trajectory: upward and downward. The internal structure of the scale is composed of two subfactors (upward and downward), and it showed good fit indices. The results from Studies 1–2 showed a good convergent validity. In all correlations with the different constructs (attitudes toward inequality, meritocratic beliefs, justification of the economic system, and status anxiety), we found opposite effects for the two types of societal mobility (upward and downward).

On the one hand, these findings are aligned with previous approaches suggesting that both types of mobility could be considered as relatively independent constructs [23]. On the other hand, the negative relationship between upward and downward societal mobility could be an important contribution to the study of subjective social mobility and its possible consequences. Taking these results into account, these effects could be two competing effects which could suppress each other [97]. Hence, the inclusion of both types of mobility in the same model could lead to non-significant results, or suppression effect. This could shed light on a more accurate and holistic view of subjective social mobility, helping to clarify whether the two types of social mobility can be understood as opposing mechanisms of mobility.

Future studies should test, through different methodologies, whether the negative relationship between upward and downward societal mobility beliefs holds.

A further important contribution could be the opposite direction in all effects found between upward and downward societal mobility on different constructs. A possible interpretation of the above results could be related to expectations about gaining and losing status [98]. That is, upward mobility beliefs could increase individuals’ hope for increasing their status, and this could lead to support for the economic status quo and feeling less status anxiety. Furthermore, people with downward mobility beliefs could think of a probable loss of status, and this could lead to less support for the status quo and feeling more status anxiety.

Measuring social mobility beliefs can be a difficult task due to the limited consensus on its theoretical definition and the complexity of the interconnected concepts (e.g., meritocracy, Protestant work ethic, etc.). As mentioned in the introduction, some instruments focus on the study of other types of social mobility [19], while others use measures that can be tedious and difficult to solve for participants [9,56]. This research showed the importance of studying subjective social mobility according to the trajectory considering its two dimensions: upward and downward.

The strength of the correlation between mobility beliefs and different legitimizing variables (meritocratic beliefs, justification of the economic system, attitudes toward inequality) was stronger for upward (vs. downward) mobility beliefs. These results are in line with previous studies [see 52] showing the relationship between social mobility and different ideological variables and suggesting the role of upward social mobility beliefs as a possible ideological variable [see 61]. Also, our findings support the Prospect Of Upward Mobility theory [POUM; 99], which suggests that individuals are willing to accept the elevated status of the wealthy because they anticipate the possibility of themselves or their children climbing to such ranks in the future. As a result, they aim to maintain the advantages associated with their prospective economic position. These results extend the literature on social mobility and go beyond previous studies by discriminating between different upward and downward mobility effects and their consequences on *statu quo* maintenance.

Concerning status anxiety, our results show consistency with the results of Melita et al. [100]. Authors suggest that status anxiety might be more associated to downward mobility beliefs than upward mobility. One possible interpretation could be related to the characteristics of our sample. First, the literature on social classes has shown differences between the living conditions of different social classes [5]. Second, most participants self-placed themselves in intermediate positions on the social scale. Therefore, the anxious effect may be stronger when holding beliefs that imply projections of future status loss and less so when being in intermediate positions on the social scale when holding beliefs that imply projections of future status gain. This may be because participants in our study might evaluate intermediate positions on the social scale as optimal positions where good living conditions exist, holding beliefs that imply projections of future status gain.

We believe that this research makes important contributions, yet it also has some limitations. For example, the sample of our studies does not represent Spanish society in terms of some sociodemographic characteristics (e.g., subjective socio-economic status, political orientation, participants’ educational attainment). However, as Winton and Sabol [101] point out, non-representative samples are useful when studying the psychometric properties of a scale, since this type of study focuses on different measurement characteristics rather than the possible outcomes derived from the scale. The structure and validity evidence for a measuring instrument is based on the consistency between indicators and their ability to reflect expected relationships with other related constructs [102]. Although we consider that the differences between the two dimensions of social mobility beliefs could be replicated in other samples and contexts, we acknowledge the limitation of having conducted our study in one specific context. Future studies should investigate whether the social mobility beliefs scale retains its psychometric properties in other circumstances and cultural contexts.

Based on our results, we believe that the development of this new instrument can help to deepen our understanding of the psychosocial consequences of subjective social mobility, as well as to differentiate two processes that may have different consequences. For instance, social mobility beliefs may be related to social income comparisons [e.g., 103]: it is likely that people who think they will go down will compare themselves to those behind them, while those who think they will go up will tend to compare upwards. Also, it may be important to explore whether there are cross-cultural differences on these beliefs [e.g., 104]. For instance, it may be argued that in individualistic countries low upward mobility may be more consequential, potentially leading to increased levels of status anxiety. Furthermore, it would also be interesting to study whether upward social mobility beliefs (vs. downward beliefs) could be a way to operationalize ideology. From this perspective, it could moderate the effects of perceived inequality on several social and psychological outcomes [40].

Finally, although we consider that the differences raised between the two dimensions of social mobility beliefs could be replicated in other samples and context, we also acknowledge the limitation of having conducted the study in a specific context. Future studies should test whether the BSMBS maintains its psychometric properties in other circumstances and cultural contexts. It would also be useful to conduct experimental manipulations on different types of mobility beliefs to test causal relationships between upward and downward mobility beliefs and their possible psychosocial effects.

## Supporting information

S1 Table Sociodemographic characteristics (Studies 1–2). (DOCX)

S2 Table Loadings of bidimensional social mobility beliefs scale and social mobility beliefs scale (Study 1). (DOCX)

S3 Table Standardized loadings based upon Polychoric correlation matrix. (DOCX)

S4 Table Coefficients and bootstrapped confidence intervals based upon Pearson correlation matrix. (DOCX)

S5 Table Interfactor correlations and bootstrapped confidence intervals. (DOCX)

S6 Table Fit indices for measurement invariance across gender and subjective socioeconomic status (Study 2). (DOCX)

S7 Table Fit indices for measurement invariance across subjective socioeconomic status (SSS). (DOCX)

S8 Table Normality tests (Study 2). (DOCX)

S9 Table Henze-Zirkler’s multivariate normality test (Study 2). (DOCX)

S10 Table Descriptive statistics of items (Study 2). (DOCX)

S11 Table Societal objective indicators for Spain. (DOCX)

S1 Appendix Bidimensional social mobility beliefs scale. (DOCX)

S1 File Panel of expert’s procedure. (DOCX)

S2 File Bidimensional social mobility beliefs scale (20 items). (DOCX)

S3 File Analyses pre-registered hypotheses (Study 2). (DOCX)
