# Supplementary material for: Rising and falling on the social ladder: The bidimensional social mobility beliefs scale
Source: PLoS One. 2023 Dec 5;18(12):e0294676. doi: 10.1371/journal.pone.0294676 (PMC10697514; doi:10.1371/journal.pone.0294676)
Supplement: S1 Table — (DOCX) [file pone.0294676.s001.docx]

**S3**

| **S1 Table. Sociodemographic Characteristics (Studies 1-2)** | | | | |  |
| --- | --- | --- | --- | --- | --- |
| Variables | **Study 1** | |  | **Study 2** |  |
|  | N = 164 | |  | N = 400 |  |
| **Age** |  | 43.41 (12.34)^1^ | 32.50 (14.05)^1^ | |  |
| **Participant’ Income** |  | €5064.32 (12172.53) | €2863.01 (5879.58) | |  |
| **Gender** |  |  |  | |  |
| Male |  | 84 (51.22%)^2^ | 153 (38.25%)^2^ | |  |
| Female |  | 78 (47.56%) | 243 (60.75%) | |  |
| Other |  | 2 (1.22%) | 4 (1.00%) | |  |
| **Marital Status** |  |  |  | |  |
| Single |  | 35 (21.34%) | 170 (42.50%) | |  |
| With partner |  | 51 (31.10%) | 130 (32.50%) | |  |
| Married |  | 74 (45.12%) | 86 (21.50%) | |  |
| Divorced |  | 3 (1.83%) | 14 (3.50%) | |  |
| Widowed |  | 1 (0.61%) | 0 (0.00%) | |  |
| **Educational Attainment** |  |  |  | |  |
| No schooling |  | 0 (0.00%) | 0 (0.00%) | |  |
| Primary education |  | 3 (1.83%) | 3 (0.75%) | |  |
| Secondary education obligatory |  | 2 (1.22%) | 4 (1.00%) | |  |
| Secondary education no obligatory |  | 11 (6.71%) | 128 (32.00%) | |  |
| Professional training |  | 16 (9.76%) | 31 (7.75%) | |  |
| University studies |  | 64 (39.02%) | 123 (30.75%) | |  |
| Postgraduate |  | 68 (41.46%) | 111 (27.75%) | |  |
| **Occupation** |  |  |  | |  |
| Unemployed |  | 15 (9.20%) | 12 (3.00%) | |  |
| Student |  | 7 (4.29%) | 163 (40.75%) | |  |
| Student and part-time worker |  | 6 (3.68%) | 35 (8.75%) | |  |
| Part-time worker |  | 3 (1.84%) | 6 (1.50%) | |  |
| Full-time worker |  | 123 (75.46%) | 176 (44.00%) | |  |
| Retired |  | 9 (5.52%) | 8 (2.00%) | |  |
| (Missing) |  | 1 | 2 (0.50%) | |  |
| **Subjective Socio-economic Status** |  |  |  | |  |
| 1 |  | 1 (0.61%) | 7 (1.75%) | |  |
| 3 |  | 8 (4.88%) | 14 (3.50%) | |  |
| 4 |  | 22 (13.41%) | 45 (11.25%) | |  |
| 5 |  | 24 (14.63%) | 88 (22.00%) | |  |
| 6 |  | 45 (27.44%) | 120 (30.00%) | |  |
| 7 |  | 46 (28.05%) | 94 (23.50%) | |  |
| 8 |  | 15 (9.15%) | 28 (7.00%) | |  |
| 9 |  | 3 (1.83%) | 2 (0.50%) | |  |
| 10 |  | 0 (0.00%) | 0 (0.00%) | |  |
| **Political Orientation** |  |  |  | |  |
| Far-left |  | 16 (9.94%) | 35 (8.79%) | |  |
| Left |  | 74 (45.96%) | 191 (47.99%) | |  |
| Center-left |  | 29 (18.01%) | 85 (21.36%) | |  |
| Center |  | 26 (16.15%) | 44 (11.06%) | |  |
| Center-right |  | 10 (6.21%) | 29 (7.29%) | |  |
| Right |  | 6 (3.73%) | 13 (3.27%) | |  |
| Far-right |  | 0 (0.00%) | 1 (0.3%) | |  |
| (Missing) |  | 3 | 2 | |  |
| *Note*: N, Total sample size; ^1^Mean (SD); ^2^Total number of participants (%) | | | | |  |
