# Supplementary material for: Rising and falling on the social ladder: The bidimensional social mobility beliefs scale
Source: PLoS One. 2023 Dec 5;18(12):e0294676. doi: 10.1371/journal.pone.0294676 (PMC10697514; doi:10.1371/journal.pone.0294676)
Supplement: S2 Table — (DOCX) [file pone.0294676.s002.docx]

**S4**

| **S2 Table. Loadings of Bidimensional Social Mobility Beliefs Scale and Social Mobility Beliefs Scale (Study 1)** | | | | |
| --- | --- | --- | --- | --- |
| Items | F1 | F2 | F3 | *h^2^* |
| SMBS_7 | -0.82 |  |  | 0.62 |
| SMBS_5 | 0.75 |  |  | 0.61 |
| SMBS_8 | -0.74 |  |  | 0.64 |
| SMBS_2 | 0.73 |  |  | 0.64 |
| SMBS_1 | -0.70 |  |  | 0.58 |
| SMBS_6 | 0.62 |  |  | 0.40 |
| SMBS_4 | 0.62 |  |  | 0.57 |
| SMBS_3 | -0.50 |  |  | 0.29 |
| BSMBS_8u |  | 0.74 |  | 0.63 |
| BSMBS_9u |  | 0.70 |  | 0.65 |
| BSMBS_4u |  | 0.65 |  | 0.53 |
| BSMBS_10u |  | 0.58 | -0.30 | 0.60 |
| BSMBS_18d |  |  | 0.72 | 0.59 |
| BSMBS_13d |  |  | 0.67 | 0.50 |
| BSMBS_14d |  |  | 0.65 | 0.52 |
| BSMBS_11d |  |  | 0.61 | 0.47 |
| *Note*: N = 164; BSMBS, Bidimensional Social Mobility Beliefs Scale; SMBS, Social Mobility Beliefs Scale; F, factor; *h*^2^, communality; Standardized loadings > .30 are reported | | | | |
