# Supplementary material for: Rising and falling on the social ladder: The bidimensional social mobility beliefs scale
Source: PLoS One. 2023 Dec 5;18(12):e0294676. doi: 10.1371/journal.pone.0294676 (PMC10697514; doi:10.1371/journal.pone.0294676)
Supplement: S3 Table — (DOCX) [file pone.0294676.s003.docx]

**S5**

| **S3 Table. Standardized loadings (pattern matrix) based upon Polychoric correlation matrix** | | | |
| --- | --- | --- | --- |
|  |  |  |  |
|  | F1 | F2 | h2 |
| BSMBS_8u | 0.86 |  | 0.65 |
| BSMBS_4u | 0.78 |  | 0.60 |
| BSMBS_9u | 0.72 |  | 0.63 |
| BSMBS_10u | 0.71 |  | 0.66 |
| BSMBS_13d |  | 0.84 | 0.62 |
| BSMBS_11d |  | 0.70 | 0.52 |
| BSMBS_14d |  | 0.69 | 0.55 |
| BSMBS_18d |  | 0.67 | 0.58 |
| *Note*: F, factor; h2, communality; Standardized loadings > .30 are reported | | | |
|  |  |  |  |
