# Supplementary material for: Rising and falling on the social ladder: The bidimensional social mobility beliefs scale
Source: PLoS One. 2023 Dec 5;18(12):e0294676. doi: 10.1371/journal.pone.0294676 (PMC10697514; doi:10.1371/journal.pone.0294676)
Supplement: S4 Table — (DOCX) [file pone.0294676.s004.docx]

**S6**

| **S4 Table**. **Coefficients and bootstrapped confidence intervals based upon Pearson correlation matrix** | | | | | | |
| --- | --- | --- | --- | --- | --- | --- |
|  |  |  |  |  |  |  |
|  | low | F1 | upper | low | F2 | upper |
| BSMBS_4u | 0.55 | 0.74 | 0.92 | -0.19 | -0.01 | 0.11 |
| BSMBS_8u | 0.74 | 0.85 | 0.93 | -0.04 | 0.12 | 0.23 |
| BSMBS_9u | 0.56 | 0.71 | 0.86 | -0.26 | -0.11 | 0.02 |
| BSMBS_10u | 0.49 | 0.67 | 0.86 | -0.36 | -0.17 | -0.01 |
| BSMBS_11d | -0.30 | -0.02 | 0.21 | 0.44 | 0.69 | 0.90 |
| BSMBS_13d | -0.16 | 0.08 | 0.25 | 0.58 | 0.79 | 0.94 |
| BSMBS_14d | -0.27 | -0.06 | 0.13 | 0.46 | 0.69 | 0.90 |
| BSMBS_18d | -0.36 | -0.13 | 0.08 | 0.41 | 0.65 | 0.88 |
| *Note*: Standardized loadings; Bootstrap = 5000; F, factor. | | | | | | |
