# Supplementary material for: Rising and falling on the social ladder: The bidimensional social mobility beliefs scale
Source: PLoS One. 2023 Dec 5;18(12):e0294676. doi: 10.1371/journal.pone.0294676 (PMC10697514; doi:10.1371/journal.pone.0294676)
Supplement: S5 Table — (DOCX) [file pone.0294676.s005.docx]

| **S5 Table. Interfactor correlations and bootstrapped confidence intervals** | | | |
| --- | --- | --- | --- |
|  |  |  |  |
|  | lower | estimate | upper |
| F1-F2 | -0.69 | -0.58 | -0.38 |
| *Note*: F, factor. | | | |
