# Supplementary material for: Rising and falling on the social ladder: The bidimensional social mobility beliefs scale
Source: PLoS One. 2023 Dec 5;18(12):e0294676. doi: 10.1371/journal.pone.0294676 (PMC10697514; doi:10.1371/journal.pone.0294676)
Supplement: S6 Table — (DOCX) [file pone.0294676.s006.docx]

**S7**

| **S6 Table.** **Fit Indices for Measurement Invariance Across Gender** | | | | | | | |
| --- | --- | --- | --- | --- | --- | --- | --- |
| Invariance | Chisq | df | pvalue | CFI | TLI | SRMR | RMSEA [90% CI] |
| Configural | 64.633 | 38 | 0.004 | 0.98 | 0.98 | 0.04 | .04 (.03, .08) |
| Metric | 70.31 | 44 | 0.007 | 0.99 | 0.98 | 0.04 | .03 (.02, .07) |
| Scalar | 76.837 | 50 | 0.009 | 0.99 | 0.98 | 0.04 | .03 (.02, .07) |
| Residual | 89.397 | 58 | 0.005 | 0.99 | 0.99 | 0.05 | .03 (.02, .07) |
| *Note*: N=400; Gender: “1” = Male; “2” = Female; CFI = Comparative fit index; TLI = Tucker-Lewis index; SRMR = Standardized Root Mean Square Residual; RMSEA = root-mean-square error of approximation; CI = confidence interval. | | | | | | | |
|  |  |  |  |  |  |  |  |
