# Supplementary material for: Rising and falling on the social ladder: The bidimensional social mobility beliefs scale
Source: PLoS One. 2023 Dec 5;18(12):e0294676. doi: 10.1371/journal.pone.0294676 (PMC10697514; doi:10.1371/journal.pone.0294676)
Supplement: S7 Table — (DOCX) [file pone.0294676.s007.docx]

| **S7 Table. Fit Indices for Measurement Invariance Across Subjective Socioeconomic Status (SSS)** | | | | | | | |
| --- | --- | --- | --- | --- | --- | --- | --- |
| Invariance | Chisq | df | pvalue | CFI | TLI | SRMR | RMSEA [90% CI] |
| Configural | 57.672 | 38 | 0.021 | 0.99 | 0.99 | 0.03 | 0.03 (.02, .07) |
| Metric | 61.751 | 44 | 0.04 | 0.99 | 0.99 | 0.04 | 0.02 (.01, .07) |
| Scalar | 75.623 | 50 | 0.011 | 0.98 | 0.98 | 0.04 | 0.03 (.02, .07) |
| Residual | 97.026 | 58 | 0.001 | 0.98 | 0.98 | 0.05 | 0.04 (.03, .07) |
| *Note*: N=400; SSS: “≤ 5” = Low SSS; “≥ 6” = High SSS; CFI = Comparative fit index; TLI = Tucker-Lewis index; SRMR = Standardized Root Mean Square Residual; RMSEA = root-mean-square error of approximation; CI = confidence interval. | | | | | | | |
|  |  |  |  |  |  |  |  |
