# Supplementary material for: Rising and falling on the social ladder: The bidimensional social mobility beliefs scale
Source: PLoS One. 2023 Dec 5;18(12):e0294676. doi: 10.1371/journal.pone.0294676 (PMC10697514; doi:10.1371/journal.pone.0294676)
Supplement: S8 Table — (DOCX) [file pone.0294676.s008.docx]

**S8**

| **S8 Table. Anderson-Darling's Univariate Normality Test (Study 2)** | | | |
| --- | --- | --- | --- |
| Variable | Statistic | p value | Normality |
| BSMBS_4u_I1 | 108.015 | <0.001 | NO |
| BSMBS_8u_I2 | 106.213 | <0.001 | NO |
| BSMBS_9u_I3 | 114.071 | <0.001 | NO |
| BSMBS_10u_I4 | 108.954 | <0.001 | NO |
| BSMBS_11d_I5 | 96.250 | <0.001 | NO |
| BSMBS_13d_I6 | 102.278 | <0.001 | NO |
| BSMBS_14d_I7 | 100.492 | <0.001 | NO |
| BSMBS_18d_I8 | 112.657 | <0.001 | NO |
| *Note*: N = 400 | | | |
