# Supplementary material for: Rising and falling on the social ladder: The bidimensional social mobility beliefs scale
Source: PLoS One. 2023 Dec 5;18(12):e0294676. doi: 10.1371/journal.pone.0294676 (PMC10697514; doi:10.1371/journal.pone.0294676)
Supplement: S9 Table — (DOCX) [file pone.0294676.s009.docx]

| **S9 Table. Henze-Zirkler's Multivariate Normality Test (Study 2)** | | |
| --- | --- | --- |
| Statistic | p value | Normality |
| 2.81 | 0 | NO |
| *Note*: N = 400 | | |
