# Supplementary material for: Rising and falling on the social ladder: The bidimensional social mobility beliefs scale
Source: PLoS One. 2023 Dec 5;18(12):e0294676. doi: 10.1371/journal.pone.0294676 (PMC10697514; doi:10.1371/journal.pone.0294676)
Supplement: S10 Table — (DOCX) [file pone.0294676.s010.docx]

**S9**

| **S10 Table. Descriptive Statistics of Items (Study 2)** | | | | | |
| --- | --- | --- | --- | --- | --- |
| Item label | M | SD | Skewness | Kurtosis | Citc |
| **Upward social mobility** |  |  |  |  |  |
| BSMBS_4u (I1) | 3.83 | 1.37 | -0.156 | -0.754 | 0.81 |
| BSMBS_8u (I2) | 3.56 | 1.36 | -0.121 | -0.754 | 0.75 |
| BSMBS_9u (I3) | 4.06 | 1.34 | -0.357 | -0.362 | 0.58 |
| BSMBS_10u (I4) | 3.96 | 1.40 | -0.180 | -0.635 | 0.67 |
| **Downward social mobility** |  |  |  |  |  |
| BSMBS_11d (I5) | 3.81 | 1.51 | 0.242 | -0.775 | 0.64 |
| BSMBS_13d (I6) | 3.25 | 1.36 | 0.429 | -0.167 | 0.66 |
| BSMBS_14d (I7) | 3.46 | 1.36 | 0.328 | -0.383 | 0.70 |
| BSMBS_18d (I8) | 3.20 | 1.29 | 0.430 | -0.130 | 0.65 |
| *Note*: N = 400; M, mean; SD, standard deviation; Citc, corrected item­-total correlation | | | | | |
