# Supplementary material for: Rising and falling on the social ladder: The bidimensional social mobility beliefs scale
Source: PLoS One. 2023 Dec 5;18(12):e0294676. doi: 10.1371/journal.pone.0294676 (PMC10697514; doi:10.1371/journal.pone.0294676)
Supplement: S11 Table — (DOCX) [file pone.0294676.s011.docx]

**S11**

| **S11 Table. Societal Objective Indicators** | | | | | | |  |  |
| --- | --- | --- | --- | --- | --- | --- | --- | --- |
|  | GINI (2020) | P50P10 (2019) | P90P10 (2019) | P90P50 (2019) | S80S20 (2019) | PALMA (2019) | HDI (2021) | IGM (2021) |
| Spain | 34.9 | 2.4 | 4.8 | 2 | 5.7 | 1.19 | 0.91 | 0.78 |
| *Note*: GINI, objective inequality index (from The World Bank, 2022); P50P10; interdecil P50/P10 (from OECD data, 2019); P90/P10, interdecil P90/P10 (from OECD data, 2019); P90/P50, interdecil P90/P50 (from OECD data, 2019); S80/S20, quintile share S80/S20 (from OECD data, 2019); PALMA, objective inequality index (from OECD data, 2019); HDI, Human Development Index (from Our World in Data, 2022); IGM, Intergenerational Mobility Index (from Our World in Data, 2021) | | | | | | | | |
|  |  |  |  |  |  |  |  |  |
|  |  |  |  |  |  |  |  |  |
|  |  |  |  |  |  |  |  |  |
