# Supplementary material for: Rising and falling on the social ladder: The bidimensional social mobility beliefs scale
Source: PLoS One. 2023 Dec 5;18(12):e0294676. doi: 10.1371/journal.pone.0294676 (PMC10697514; doi:10.1371/journal.pone.0294676)
Supplement: S1 Appendix — (DOCX) [file pone.0294676.s012.docx]

**S12**

**S1 Appendix**. **Bidimensional Social Mobility Beliefs Scale (BSMBS)**

**Upward social mobility dimension:**

- Item 1 (BSMBS_4u): En España, es frecuente que los(as) hijos(as) consigan un estatus socioeconómico superior al del hogar en el que crecieron (In Spain, children often achieve a higher socioeconomic status than the household in which they grew up).
- Item 2 (BSMBS_8u): Los/as hijos/as de las personas españolas llegan a pertenecer a una clase social más alta en comparación con la clase de la que provienen (The children of Spanish people come to belong to a higher social class compared to the class they come from).
- Item 3 (BSMBS_9u): La mayoría de la población española mejora su estatus socioeconómico a lo largo de su vida (The majority of the Spanish population improves their socioeconomic status throughout their lives).
- Item 4 (BSMBS_10u): Generalmente, en España, los(as) hijos(as) tienen mejores puestos de trabajo de una generación a otra (In Spain, children in general have better jobs from one generation to the next).

**Downward social mobility dimension:**

- Item 5 (BSMBS_11d): En la sociedad española, la mayoría de las personas tienen ingresos más bajos de una generación a otra (In Spanish society, most people have lower incomes from one generation to the next).
- Item 6 (BSMBS_13d): La mayoría de las familias españolas ocupan posiciones sociales inferiores a las de la generación anterior (The majority of Spanish families have lower social positions than the previous generation).
- Item 7 (BSMBS_14d): En España, es frecuente que los(as) hijos(as) consigan un estatus socioeconómico inferior al del hogar en el que crecieron (In Spain, children often achieve a lower socioeconomic status than the household in which they grew up).
- Item 8 (BSMBS_18d): Los/as hijos/as de las personas españolas llegan a pertenecer a una clase social más baja en comparación con la clase de la que provienen (The children of Spanish people come to belong to a lower social class compared to the class they come from).
