# Supplementary material for: Rising and falling on the social ladder: The bidimensional social mobility beliefs scale
Source: PLoS One. 2023 Dec 5;18(12):e0294676. doi: 10.1371/journal.pone.0294676 (PMC10697514; doi:10.1371/journal.pone.0294676)
Supplement: S1 File — (DOCX) [file pone.0294676.s013.docx]

**S1**

**Panel of expert’s procedure**

Following DeVellis (2017), we created an item pool at least three times as large as the final scale. Then, a panel of experts was selected to evaluate different dimensions of the items: ambiguity, representativeness, intelligibility, and relevance (Carretero-Dios & Pérez, 2005). The panel of experts comprised five experienced researchers in social psychology and behavioral science methodology (Lynn, 1986). A self-administered online questionnaire invited a panel of experts to evaluate the dimensions of the 26 items that made up the battery of items on social mobility beliefs. The questionnaire included, in the following order: task instructions, conceptual delimitation of the construct (social mobility) and the subdimensions (upward and downward social), the items, and different questions about the assessment of the items. The judges should indicate the *ambiguity* of the item (i.e., the category to which the item corresponded: upward or downward mobility) and evaluate it on a 5-point Likert scale (Haynes et al., 1995). Also, the *representativeness* (1 = "Not at all"; 5 = "Completely"), *intelligibility* (1 = "Not at all understandable"; 5 = "Very understandable"), and *relevance* (1 = "Unimportant"; 5 = "Very important"). Finally, some considerations and comments for improvement were collected. A content validity index (CVI) ≥ 80% was established (Hyrkäs et al., 2003). Considering the above criteria, six items were eliminated as they had insufficient content validity, that is, below the default value (CVI ≤ 80%). The resulting scale was composed of 20 items on social mobility beliefs. The items represent beliefs in upward (10 items) and downward (10 items) social mobility.
