# Supplementary material for: Rising and falling on the social ladder: The bidimensional social mobility beliefs scale
Source: PLoS One. 2023 Dec 5;18(12):e0294676. doi: 10.1371/journal.pone.0294676 (PMC10697514; doi:10.1371/journal.pone.0294676)
Supplement: S2 File — (DOCX) [file pone.0294676.s014.docx]

**S2**

**Bidimensional Social Mobility Beliefs Scale (20 items)**

**Upward social mobility dimension:**

- BSMBS_1u: En la sociedad española, la mayoría de las personas tienen ingresos más altos de una generación a otra (In Spanish society, most people have higher incomes from one generation to the next).
- BSMBS_2u: Gran parte de la población española mejora su salario a lo largo de su vida (The majority of the Spanish population improves their salaries during their lifetime).
- BSMBS_3u: La mayoría de las familias españolas ocupan posiciones sociales superiores a las de la generación anterior (Most Spanish families occupy higher social positions than those of the previous generation).
- BSMBS_4u: En España, es frecuente que los(as) hijos(as) consigan un estatus socioeconómico superior al del hogar en el que crecieron (In Spain, children often achieve a higher socio-economic status than the household in which they grew up.).
- BSMBS_5u: En la sociedad española es frecuente que, a lo largo de su vida, una persona acabe trabajando en ocupaciones de mayor prestigio (In Spanish society it is often that, over the course of a person's life, a person ends up working in occupations of higher prestige).
- BSMBS_6u: Es bastante común en la sociedad española que las personas, a lo largo de su vida, asciendan a posiciones sociales más altas (It is quite common in Spanish society for people, over the course of their lifetime, to rise to higher social positions).
- BSMBS_7u: En España, las posibilidades de que los(as) hijos(as) consigan un nivel educativo mayor al de sus padres y madres son altas (In Spain, the chances of children achieving a higher level of education than their parents are high).
- BSMBS_8u: Los/as hijos/as de las personas españolas llegan a pertenecer a una clase social más alta en comparación con la clase de la que provienen (The children of Spanish people come to belong to a higher social class compared to the class they come from).
- BSMBS_9u: La mayoría de la población española mejora su estatus socioeconómico a lo largo de su vida (The majority of the Spanish population improves their socio-economic status throughout their lifetime).
- BSMBS_10u: Generalmente, en España, los(as) hijos(as) tienen mejores puestos de trabajo de una generación a otra (In Spain, children in general have better jobs from one generation to the next).

**Downward social mobility dimension:**

- BSMBS_11d: En la sociedad española, la mayoría de las personas tienen ingresos más bajos de una generación a otra (In Spanish society, most people have lower incomes from one generation to the next.).
- BSMBS_12d: Gran parte de la población española empeora su salario a lo largo de su vida (The majority of the Spanish population worsens their salaries during their lifetime).
- BSMBS_13d: La mayoría de las familias españolas ocupan posiciones sociales inferiores a las de la generación anterior (The majority of Spanish families have lower social positions than the previous generation).
- BSMBS_14d: En España, es frecuente que los(as) hijos(as) consigan un estatus socioeconómico inferior al del hogar en el que crecieron (In Spain, children often achieve a lower socio-economic status than the household in which they grew up).
- BSMBS_15d: En la sociedad española es frecuente que, a lo largo de su vida, una persona acabe trabajando en ocupaciones de menor prestigio (In Spanish society it is often that, over the course of a person's life, a person ends up working in occupations of lower prestige).
- BSMBS_16d: Es bastante común en la sociedad española que las personas, a lo largo de su vida, desciendan a posiciones sociales más bajas (It is quite common in Spanish society for people, over the course of their lifetime, to descend to lower social positions).
- BSMBS_17d: En España, las posibilidades de que los(as) hijos(as) consigan un nivel educativo mayor al de sus padres y madres son bajas (In Spain, the chances of children achieving a higher level of education than their parents are low).
- BSMBS_18d: Los/as hijos/as de las personas españolas llegan a pertenecer a una clase social más baja en comparación con la clase de la que provienen (The children of Spanish people come to belong to a lower social class compared to the class they come from).
- BSMBS_19d: La mayoría de la población española empeora su estatus socioeconómico a lo largo de su vida (The majority of the Spanish population worsen their socio-economic status throughout their lifetime).
- BSMBS_20d: Generalmente, en España, los(as) hijos(as) tienen peores puestos de trabajo de una generación a otra (In Spain, children in general have worsen jobs from one generation to the next).
