# Supplementary material for: Rising and falling on the social ladder: The bidimensional social mobility beliefs scale
Source: PLoS One. 2023 Dec 5;18(12):e0294676. doi: 10.1371/journal.pone.0294676 (PMC10697514; doi:10.1371/journal.pone.0294676)
Supplement: S3 File — (DOCX) [file pone.0294676.s015.docx]

**S10**

**Analyses pre-registered hypotheses (Study 2)**

To test the original pre-registered hypotheses, we conducted a significance test for the comparison of correlations. The results showed significant differences between the correlations of the types of social mobility (upward and downward) and meritocratic beliefs (Z-score = 11.92; *p* ≤ .001), confirming H1, economic system justification (Z-score = 9.18; *p* ≤ .001), confirming H2, and status anxiety (Z-score = 6.23; *p* ≤ .001), confirming H3.
