# Supplementary material for: Rising and falling on the social ladder: The bidimensional social mobility beliefs scale
Source: PLoS One. 2023 Dec 5;18(12):e0294676. doi: 10.1371/journal.pone.0294676 (PMC10697514; doi:10.1371/journal.pone.0294676)
Supplement: Attachment — Submitted filename: Response to Reviewers.docx [file pone.0294676.s016.docx]

**RESPONSE TO REVIEWERS**

**Response to Reviewer 1:**

We thank you for your valuable comments and critique, which will undoubtedly strengthen the quality of our research. Your feedback helped us in shaping the new manuscript. The changes (in yellow in the manuscript) that we have introduced in the revision process are detailed below.

1. The theme of the paper is potentially relevant, however, the findings advocating for a two dimensional mobility scale are unconvincing. The main problem I see is that the announced major contribution – creating a two dimensional scale of social stratification – is somewhat uncompelling. Are upward and downward social mobility really two dimensions of social stratification or are they just opposite directions on one dimension? The correlations provided within the article seem to support this view as both “dimensions” correlate with related concepts in an opposite manner, and they are also mutually negatively correlated. I do not see compelling evidence that such: ”bi-dimensional” scale provides more explanatory power over “classical” / “uni-dimensional” scales of subjective social mobility.

The problem is clearly visible in the claims of the authors e.g. “In Study 1, our main goal was to explore whether upward and downward social mobility are two INDEPENDENT and negatively RELATED dimensions in social mobility (Objective 1).” (p. 7, CAPS added) The two “dimensions” are hardly independent as they are strongly correlated (-0.53 or -0.44). Correlations in Tables 1 and 5 show that on most occasions the upward social mobility beliefs correlate stronger with related constructs.
In this regard, I do not agree with the claim of the authors in the discussion in study 2:
“From this last perspective, it could be assumed that both types of mobility (upward and downward) have the same relationship with other variables. But we have shown that they do not: Upward is positively correlated with meritocracy and economic system justification, whereas downward is negatively correlated with it; the opposite is true when considering status anxiety. As such, the fact the correlation sign is different indicated that the variables are predicting different effects.” (p. 18).
Imagine a scale of happiness and unhappiness. The fact that happiness correlates positively and unhappiness negatively with overall life satisfaction does not mean that there is a need for a bi-dimensional scale of happiness.

R. Thank you very much for the suggestion. We appreciate the reviewer's recognition of the potential relevance of the article's topic. We believe that social stratification is a complex phenomenon, and considering it from a two-dimensional perspective will lead to a greater understanding of the topic, which may be of great value in addressing contemporary social problems. In short, we believe that the trajectory of social mobility is a bidimensional construct because of several empirical and theoretical reasons that we will elaborate on next.

First, as discussed in the manuscript, theory suggests that upward and downward social mobility beliefs can be studied as independent constructs (Browman et al., 2017; Davidai & Wienk, 2021; Day & Fiske, 2019). For example, one person could think that a given society is doing worse than before; from this perspective, they could perceive a high downward mobility (i.e., everyone is decreasing their social status), but not a high upward mobility. Other person, however, could think that a society is working in a meritocratic way; as such, he could think that there is both high upward and downward mobility at the same time: Given that people have different levels of effort and ability, they will easily move up or down in their society. In this example, we could see that there is a bidimensional nature of the construct, which does not happen with other unidimensional constructs. For instance, people cannot think that they are happy and unhappy at the same time. However, it is possible to think that —because life is a lottery or because the meritocratic system works perfectly— there is a high level of both upward and downward social mobility.

Second, we believe that there are empirical reasons for these bidimensionality. First, we found content validity evidence in favor of the bifactorial model. In Study 2, we conducted a confirmatory factor analysis that indicated that the bidimensional scale on social mobility is composed of two related factors (see Figure 1), with good psychometric properties. More importantly, in Study 2, we tested whether the scale fit indices were better adjusted for a unidimensional model. We found that only the bidimensional model had good fit indices. Therefore, this evidence supports our assumption that social mobility beliefs is a bidimensional construct.

Third, there is also external validity evidence that favors that these are two independent constructs. Across eight studies, Davidai & Gilovich (2015) found asymmetric predictions of upward and downward social mobility beliefs about performance in competitive environments. Specifically, they found that people believe they are more likely to move up the rankings than down. The authors argue that upward and downward mobility are not only two different constructs but that they are also explained by entirely different psychological mechanisms, which could be explained by people's tendency to give more importance to facilitating factors than inhibiting factors when making a judgment.

Fourth, we consider that the correlations between upward and downward social mobility beliefs found in Studies 1-2 (-0.53 and -0.44), although the correlation indices reflect a moderate relationship, can be understood as two theoretically different processes (Davidai & Wienk, 2021; Day & Fiske, 2019). The magnitude of the effect between two latent variables or factors only indicates a relationship between them, but not (necessarily) that they can be studied from a unidimensional perspective of the construct. To study how many factors or latent variables the observed items or indicators can be grouped, it would be necessary that in the item reduction technique carried out (factor analysis), the solution would have been the use of a single factor (Brown, 2015; Catena et al., 2003). In our case, the solution was two factors.

Moreover, to our knowledge, there has yet to be a consensus in the literature on the magnitude and direction of the correlation effect between upward and downward mobility beliefs. Previous studies find positive correlations between upward and downward mobility beliefs (see Browman et al., 2021), betas coefficients in the same direction (positive; see Mijs et al., 2022), or both, negative correlations between both types of mobility and betas coefficients in the same direction (see Shariff et al., 2016). Based on social mobility theory and our scale results, we consider upward and downward mobility beliefs as opposite processes. However, we agree with the reviewer when she/he argues that all this line of reasoning does not mean that “the fact the correlation sign is different indicated that the variables are predicting different effects.” (p. 18). We agree that this is not correct –as the Reviewer argues, a happiness/unhappiness scale will also have a different correlation sign, although it is the same construct. As such, we have deleted this phrase from the discussion section, and we no longer use this as evidence for the bidimensionality—although we do believe that there is a bidimensional construct, for all the other reasons described above and in the manuscript.

2. Moreover, the authors rely on data from non-random and non-representative samples. The two samples used also differ systematically. Sample 1 is basically a sample of high earning university-educated respondents, while sample 2 is notably younger and earns significantly less. Perhaps this is due to changing recruitment method which for Study 2 included “institutional mail of a university in southeast Spain.” Also, based on descriptive statistics in Table S3 both samples seem to be skewed politically towards the left, which does not seem to correspond with the overall views of the Spanish population. This bias might influence the observed views on social stratification and further limits possible generalizations.

R. Thank you very much for your comment. We agree that this could have biased the results. However, research tends to find that non-representative samples could be useful when studying the psychometric properties of a measure (see Winton & Sabol, 2022). Most (if not all) of the scales used in social psychology tend to be created using non-representative samples. But we recognize this may be a limitation of this (and of most) social psychological research studies. As such, we have included this in line 505:

“However, as Winton and Sabol [89] point out, non-representative samples are useful when studying the psychometric properties of a scale, since this type of study focuses on different measurement characteristics rather than the possible outcomes derived from the scale. The structure and validity evidence for a measuring instrument is based on the consistency between indicators and their ability to reflect expected relationships with other related constructs [90]. Although we consider that the differences between the two dimensions of social mobility beliefs could be replicated in other samples and contexts, we acknowledge the limitation of having conducted our study in one specific context. Future studies should investigate whether the social mobility beliefs scale retains its psychometric properties in other circumstances and cultural contexts”.

Also, to study a potential bias in our sample we provide a more robust of the structure and stability of our scale (see line 402). We explored the configural, metric, scalar, and residual invariance across gender (Male, Female) and Subjective Socioeconomic Status (“≤ 5” = Low SSS; “≥ 6” = High SSS). The results showed a good fit of configural, metric, scalar, and residual invariance across Gender (see Table S6), and Subjective Socioeconomic Status (see Table S7). As such, the sociodemographic differences of both samples does not influence the structure and the stability of our scale.

3. The information “Voluntary participation in the study was requested via text message.” (p. 8) is not clear. What kind of text message? Send to whom?

R. Thanks very much for your comment. We agree this sentence is not very clear, and it needs to be rewritten. To address this point, we have rewritten the sentence (see line 191):

“targeted to social network users. The message consisted of a short text encouraging participation in a study on social issues and a link to access the survey.”

4. Why are the scale items in supplement S2 only available in Spanish?

R. Thanks very much for your comment. We agree that adding a translation of the original items used in English may increase the comprehensibility of the pool of items we used in Study 1. We have added an English translation of all items in the supplementary material (see S2).

5. References do not seem to be complete. E.g. item 4 is not a full bibliographic record as it is missing the name of the journal, volume sand issue information.

R. Thanks very much for your comment. There was indeed an error when executing the reference from the reference manager. We corrected it (see line 543). We also double-check all references.

**Reviewer 2**

**Response to Reviewer 2:**

We thank you for your valuable comments and critique, which will undoubtedly strengthen the quality of our research. Your feedback helped us in shaping the new manuscript. The changes (in yellow in the manuscript) that we have introduced in the revision process are detailed below.

1. To my surprise the authors argue that “the subjective dimension of social mobility has been overlooked” and I think a significant concern with the paper is its apparent oversight of important preceding research in the domain of subjective social mobility (e.g., Shane, & Heckhausen, 2013; Weiss & Bloechl, 2023).

R. Thank you very much for the suggestion. We agree that the expression was not the most appropriate. We intended not to argue that the subjective dimension has not been studied; we just wanted to say that subjective social mobility has been studied much less than objective social mobility. We modified the statement to correct the syntactic error (see line 42).

“the subjective dimension of social mobility has been less studied than objective”

Also, we have added some references (Shane, & Heckhausen, 2013; Weiss & Bloechl, 2023) to reinforce the evidence from subjective social mobility studies (see line 57)

2. In a similar vein, it is crucial to know how the current work is positioned against or in alignment with existing models, theories, or empirical evidence. A deeper integration with prior work would have not only strengthened the foundation of the study but also situated its contributions more clearly within the broader discourse.

R. Thanks very much for your comment. We have added several paragraphs in the discussion that we believe could address this limitation (see line 479).

“The strength of the correlation between mobility beliefs and different legitimizing variables (meritocratic beliefs, justification of the economic system, attitudes toward inequality) was stronger for upward (vs. downward) mobility beliefs. These results are in line with previous studies (see Mijs et al., 2022) showing the relationship between social mobility and different ideological variables and suggesting the role of upward social mobility beliefs as a possible ideological variable (see Major et al., 2002). Also, our findings support the Prospect Of Upward Mobility theory (POUM; Benabou & Ok, 2001), which suggests that individuals are willing to accept the elevated status of the wealthy because they anticipate the possibility of themselves or their children climbing to such ranks in the future. As a result, they aim to maintain the advantages associated with their prospective economic position. These results extend the literature on social mobility and go beyond previous studies by discriminating between different upward and downward mobility effects and their consequences on *statu quo* maintenance.

Concerning status anxiety, our results show consistency with the results of Melita et al. (2023). Authors suggest that status anxiety might be more associated to downward mobility beliefs than upward mobility. One possible interpretation could be related to the characteristics of our sample. First, the literature on social classes has shown differences between the living conditions of different social classes (Manstead, 2018). Second, most participants self-placed themselves in intermediate positions on the social scale. Therefore, the anxious effect may be stronger when holding beliefs that imply projections of future status loss and less so when being in intermediate positions on the social scale when holding beliefs that imply projections of future status gain. This may be because participants in our study might evaluate intermediate positions on the social scale as optimal positions where good living conditions exist, holding beliefs that imply projections of future status gain.”

3. In addition, it would be helpful to understand why certain constructs have been selected for validation (e.g., Meritocratic Beliefs Scale; Economic System Justification Scale; Status Anxiety Scale) but not others.

R. Thanks very much for your comment. The predictive validity analyses shown in our manuscript aim to show adequate evidence of the difference between the predictive abilities of upward and downward social mobility beliefs. The different constructs were selected based on theoretical-empirical and psychometric reasons. From a theoretical-empirical perspective, meritocratic beliefs, economic system justification, and status anxiety have been related to social mobility beliefs in previous studies (Day & Fiske, 2017, 2019; Davidai & Wienk, 2021; Melita et al., 2023; Mijs et al., 2022). However, these studies do not make a clear distinction between the two possible types—upward and downward— of social mobility. From a psychometric perspective, one requirement to study the psychometric characteristics of a scale of measure is to analyze the external validity evidences of the different dimensions of the scale (Carretero-Dios & Pérez, 2005; Haladyna & Rodriguez, 2013; Hambleton, 2005; Lane et al., 2016). Given that, we tried to select different constructs that would allow us to analyze whether both types of beliefs, upward and downward, are related differently to previously related constructs in the specialized literature.

For the above, we consider that meritocratic beliefs, economic system justification, and status anxiety can provide adequate evidence to determine whether the proposed scale presents adequate predictive validity.

4. Another major shortfall in the paper is the absence of clear definitions for its primary constructs (e.g., social class). This omission might lead to ambiguity and potentially different interpretations of the study's findings.

R. Thank you very much for the suggestion. We agree that a definition for its primary constructs, such as social class, in the introductory section can facilitate understanding the framework of the present research. To address this gap, we have added a paragraph in the introduction in which we deal with this topic (see line 26):

“In objective terms, social class is usually determined by a combination of three types of resources: economic (Piff & Moskowitz, 2018), educational (Snibbe & Markus, 2005; Stephens et al., 2007), and occupational prestige (Na et al., 2018; Oakes & Rossi, 2003). However, social psychology has emphasized the importance of subjective perception when conceptualizing social class (Antonoplis, 2023; Manstead, 2018; Kraus et al., 2009). In this way, the psychosocial perspective takes as a reference previous studies carried out in other fields of social sciences and defines *social class* as “a stratification system based on access to resources such as wealth, property, power, and prestige (Moya & Fiske, 2017, p. 9).”

5. The authors used twenty items chosen by experts but didn't clarify the criteria for expert selection, leaving questions about their qualifications. The process behind item selection remains vague, with no insight into expert deliberations or refinement of initial choices. Additionally, the reasoning for choosing certain items over others is missing. This lack of detail hinders understanding and contextualizing the study's foundations.

R. Thanks for the comment. In order to be as concise as possible without affecting the comprehension and understanding of the process of our research, we decided to move the section on expert judgment to supplementary material (see S1). The section on the Expert Panel Procedure includes information on the procedure carried out and the structure of the questionnaire. In particular, task instructions, conceptual delimitation of the construct (social mobility) and the subdimensions (upward and downward social), the items, and different questions about the assessment of the items: ambiguity of the item, representativeness intelligibility, and relevance. We also report that the criterion followed for selecting the items was based on the content validity index and the cut-off point selected according to the specialized literature (see Hyrkäs et al., 2003). However, we agree that the writing has room for improvement for greater understanding. To improve the text and clearly present the expert panel procedure, we have rewritten section S1 (see S1 in supplementary material).

“Following DeVellis (2017), we created an item pool at least three times as large as the final scale. Then, a panel of experts was selected to evaluate different dimensions of the items: ambiguity, representativeness, intelligibility, and relevance (Carretero-Dios & Pérez, 2005). The panel of experts comprised five experienced researchers in social psychology and behavioral science methodology (Lynn, 1986). A self-administered online questionnaire invited a panel of experts to evaluate the dimensions of the 26 items that made up the battery of items on social mobility beliefs. The questionnaire included, in the following order: task instructions, conceptual delimitation of the construct (social mobility) and the subdimensions (upward and downward social), the items, and different questions about the assessment of the items. The judges should indicate the *ambiguity* of the item (i.e., the category to which the item corresponded: upward or downward mobility) and evaluate it on a 5-point Likert scale (Haynes et al., 1995). Also, the *representativeness* (1 = "Not at all"; 5 = "Completely"), *intelligibility* (1 = "Not at all understandable"; 5 = "Very understandable"), and *relevance* (1 = "Unimportant"; 5 = "Very important"). Finally, some considerations and comments for improvement were collected. A content validity index (CVI) ≥ 80% was established (Hyrkäs et al., 2003). Considering the above criteria, six items were eliminated as they had insufficient content validity, that is, below the default value (CVI ≤ 80%). The resulting scale was composed of 20 items on social mobility beliefs. The items represent beliefs in upward (10 items) and downward (10 items) social mobility.”

6. Finally, the discussion is underdeveloped and only frequently restates the differentiation between upward and downward mobility without significantly deepening the reader's understanding. The assertions about these mobility beliefs' implications, while intriguing, are presented without adequate exploration or substantiation. Unfortunately, the discussion does not robustly integrate or juxtapose its findings within the wider framework of social psychology or societal mobility literature.

R. Thanks very much for your comment. To address this comment, we have added a deeper explanation in the discussion section (see line 479):

“The strength of the correlation between mobility beliefs and different legitimizing variables (meritocratic beliefs, justification of the economic system, attitudes toward inequality) was stronger for upward (vs. downward) mobility beliefs. These results are in line with previous studies (see Mijs et al., 2022) showing the relationship between social mobility and different ideological variables and suggesting the role of upward social mobility beliefs as a possible ideological variable (see Major et al., 2002). Also, our findings support the Prospect Of Upward Mobility theory (POUM; Benabou & Ok, 2001), which suggests that individuals are willing to accept the elevated status of the wealthy because they anticipate the possibility of themselves or their children climbing to such ranks in the future. As a result, they aim to maintain the advantages associated with their prospective economic position. These results extend the literature on social mobility and go beyond previous studies by discriminating between different upward and downward mobility effects and their consequences on *statu quo* maintenance.

Concerning status anxiety, our results show consistency with the results of Melita et al. (2023). Authors suggest that status anxiety might be more associated to downward mobility beliefs than upward mobility. One possible interpretation could be related to the characteristics of our sample. First, the literature on social classes has shown differences between the living conditions of different social classes (Manstead, 2018). Second, most participants self-placed themselves in intermediate positions on the social scale. Therefore, the anxious effect may be stronger when holding beliefs that imply projections of future status loss and less so when being in intermediate positions on the social scale when holding beliefs that imply projections of future status gain. This may be because participants in our study might evaluate intermediate positions on the social scale as optimal positions where good living conditions exist, holding beliefs that imply projections of future status gain.”

**Reviewer 3**

**Response to Reviewer 3:**

1. Your research brings novelty, rigor and a new needed measure. You did a magnificent work with superb conceptual and methodological clarity.
This research could be targeted as an example of elegance and simplicity within a complex topic. My congratulations

R. Thank you for your comment. We are pleased to hear that you have enjoyed reading our work.
